# P-1639. Assessing Disparities in Inappropriate Outpatient Antibiotic Prescriptions in Tennessee

**DOI:** 10.1093/ofid/ofae631.1805

**Published:** 2025-01-29

**Authors:** Katie A Thure, Glodi Mutamba, Erika Kirtz, Christopher D Evans

**Affiliations:** Tennessee Department of Health, Nashville, Tennessee; Tennessee Department of Health, Nashville, Tennessee; Tennessee Department of Health, Nashville, Tennessee; Tennessee Department of Health, Nashville, Tennessee

## Abstract

**Background:**

The Centers for Disease Control and Prevention (CDC) estimates almost 47 million antibiotics are prescribed each year for indications that do not warrant antibiotic use. Identifying populations that are inappropriately prescribed antibiotics is imperative to address health inequities and prevent antimicrobial resistance. Tennessee Department of Health (TDH) evaluated disparities in outpatient antibiotic prescriptions in patients of different age groups, gender, geographical location, and insurance type.

**Methods:**

The IQVIA Medical Claims and Longitudinal Prescription Claims databases were used to perform a cross-sectional study to evaluate the appropriateness of all outpatient antibiotics dispensed from retail pharmacies in TN from January 1–December 31, 2022. Data were categorized using a tiered system utilizing ICD-10 codes. Logistic regression was performed to predict whether age, gender, insurance type, and residence were associated with receiving an antibiotic in encounters for which antibiotics are rarely indicated (tier 3).

**Results:**

In 2022, 2,017,863 prescribed antibiotics were linked to the medical claims database. Over half of these antibiotics were classified as a tier 3 encounter. Females were prescribed more antibiotics (62.0%). For tier 3 encounters specifically: 41.0% of antibiotics were prescribed to patients 65 and older. Residence in low and high socially vulnerable index (SVI) counties accounted for 27.9% and 24.9% of antibiotics, respectively. 37.2%, 16.5%, and 44.5% of antibiotics prescribed were billed to third-party payers (*e.g*., private health insurance), Medicaid, and Medicare Part D, respectively. Logistic regression showed female gender, younger age, and residence in low SVI had significantly higher odds of receiving an antibiotic for a tier 3 encounter; whereas prescriptions filled with Medicaid and Medicare were less likely to be associated with tier 3 encounters.

**Conclusion:**

Previously TDH had not explored outpatient antibiotic use through a health equity lens. While the study provided basic demographic information about patients, there is a need to continue exploring disparities within various populations to aid in targeting inventions that drive appropriate antimicrobial use in ambulatory clinical settings.

**Disclosures:**

**All Authors**: No reported disclosures

